# Biodiversity Loss Threatens Human Well-Being

**DOI:** 10.1371/journal.pbio.0040277

**Published:** 2006-08-15

**Authors:** Sandra Díaz, Joseph Fargione, F. Stuart Chapin, David Tilman

## Abstract

Biodiversity lies at the core of ecosystem processes fueling our planet's vital life-support systems; its degradation--by us--is threatening our own well-being and will disproportionately impact the poor.

The diversity of life on Earth is dramatically affected by human alterations of ecosystems [ 1]. Compelling evidence now shows that the reverse is also true: biodiversity in the broad sense affects the properties of ecosystems and, therefore, the benefits that humans obtain from them. In this article, we provide a synthesis of the most crucial messages emerging from the latest scientific literature and international assessments of the role of biodiversity in ecosystem services and human well-being.

Human societies have been built on biodiversity. Many activities indispensable for human subsistence lead to biodiversity loss, and this trend is likely to continue in the future. We clearly benefit from the diversity of organisms that we have learned to use for medicines, food, fibers, and other renewable resources. In addition, biodiversity has always been an integral part of the human experience, and there are many moral reasons to preserve it for its own sake. What has been less recognized is that biodiversity also influences human well-being, including the access to water and basic materials for a satisfactory life, and security in the face of environmental change, through its effects on the ecosystem processes that lie at the core of the Earth's most vital life support systems ( Figure 1).

**Figure 1:**
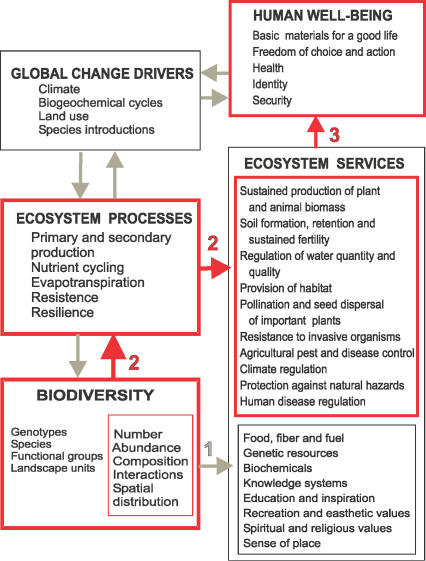
Biodiversity Is Both a Response Variable Affected by Global Change Drivers and a Factor That Affects Human Well-Being Links developed in this article are indicated in red. In the biodiversity box, the hierarchical components of biodiversity (genotypes, species, functional groups, and landscape units) each have the characteristics listed in the sub-box and explained in Figure 2 (number, relative abundance, composition, spatial distribution, and interactions involved in “vertical” diversity). Modified from [ 3, 4].

**Figure 2:**
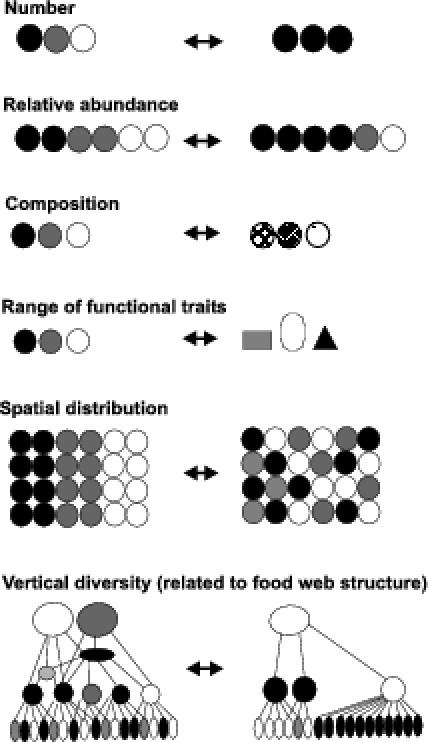
The Different Components of Biodiversity All of these components can be affected by human intervention (arrows), and in turn have repercussions for ecosystem properties and services. Symbols represent individuals or biomass units. Symbols of different shades represent different genotypes, phenotypes, or species.

Three recent publications from the Millennium Ecosystem Assessment [ 2–4], an initiative involving more than 1,500 scientists from all over the world [ 5], provide an updated picture of the fundamental messages and key challenges regarding biodiversity at the global scale. Chief among them are: (a) human-induced changes in land cover at the global scale lead to clear losers and winners among species in biotic communities; (b) these changes have large impacts on ecosystem processes and, thus, human well-being; and (c) such consequences will be felt disproportionately by the poor, who are most vulnerable to the loss of ecosystem services.

## What We Do Know: Functional Traits Matter Most

Biodiversity in the broad sense is the number, abundance, composition, spatial distribution, and interactions of genotypes, populations, species, functional types and traits, and landscape units in a given system ( Figure 2). Biodiversity influences ecosystem services, that is, the benefits provided by ecosystems to humans, that contribute to making human life both possible and worth living [ 4] ( Box 1). As well as the direct provision of numerous organisms that are important for human material and cultural life ( Figure 1, path 1), biodiversity has well-established or putative effects on a number of ecosystem services mediated by ecosystem processes ( Figure 1, path 2). Examples of these services are pollination and seed dispersal of useful plants, regulation of climatic conditions suitable to humans and the animals and plants they consider important, the control of agricultural pests and diseases, and the regulation of human health. Also, by affecting ecosystem processes such as biomass production by plants, nutrient and water cycling, and soil formation and retention, biodiversity indirectly supports the production of food, fiber, potable water, shelter, and medicines. The links between biodiversity and ecosystem services have been gaining increasing attention in the scientific literature of the past few years [ 2–4, 6]. However, not until now has there been an effort to summarize those components of biodiversity that do, or should, matter the most for the provision of these services, and the underlying mechanisms explaining those links ( Table 1; see also [ 3]).

**Table 1:**
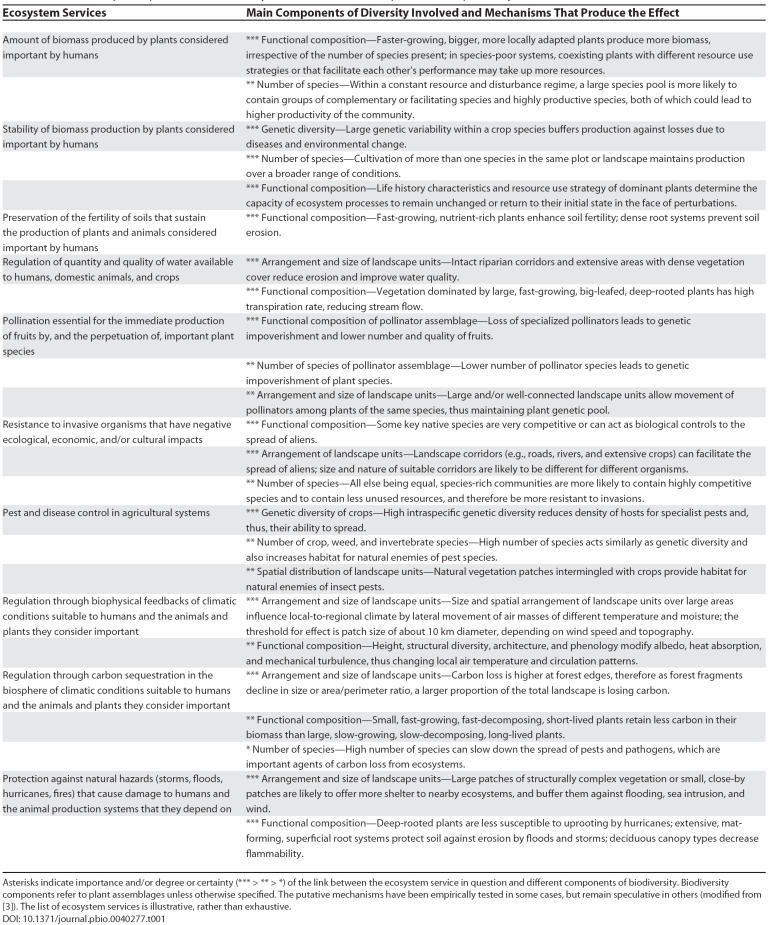
Biodiversity Components Affect Ecosystem Services in Multiple and Complex Ways

A few key messages can be drawn from existing theory and empirical studies. The first is that the number and strength of mechanistic connections between biodiversity and ecosystem processes and services clearly justify the protection of the biotic integrity of existing and restored ecosystems and its inclusion in the design of managed ecosystems. All components of biodiversity, from genetic diversity to the spatial arrangement of landscape units, may play a role in the long-term provision of at least some ecosystem services. However, some of these components are more important than others in influencing specific ecosystem services. The evidence available indicates that it is functional composition—that is, the identity, abundance, and range of species traits—that appears to cause the effects of biodiversity on many ecosystem services. At least among species within the same trophic level (e.g., plants), rarer species are likely to have small effects at any given point in time. Thus, in natural systems, if we are to preserve the services that ecosystems provide to humans, we should focus on preserving or restoring their biotic integrity in terms of species composition, relative abundance, functional organization, and species numbers (whether inherently species-poor or species-rich), rather than on simply maximizing the number of species present.

**Figure 3:**
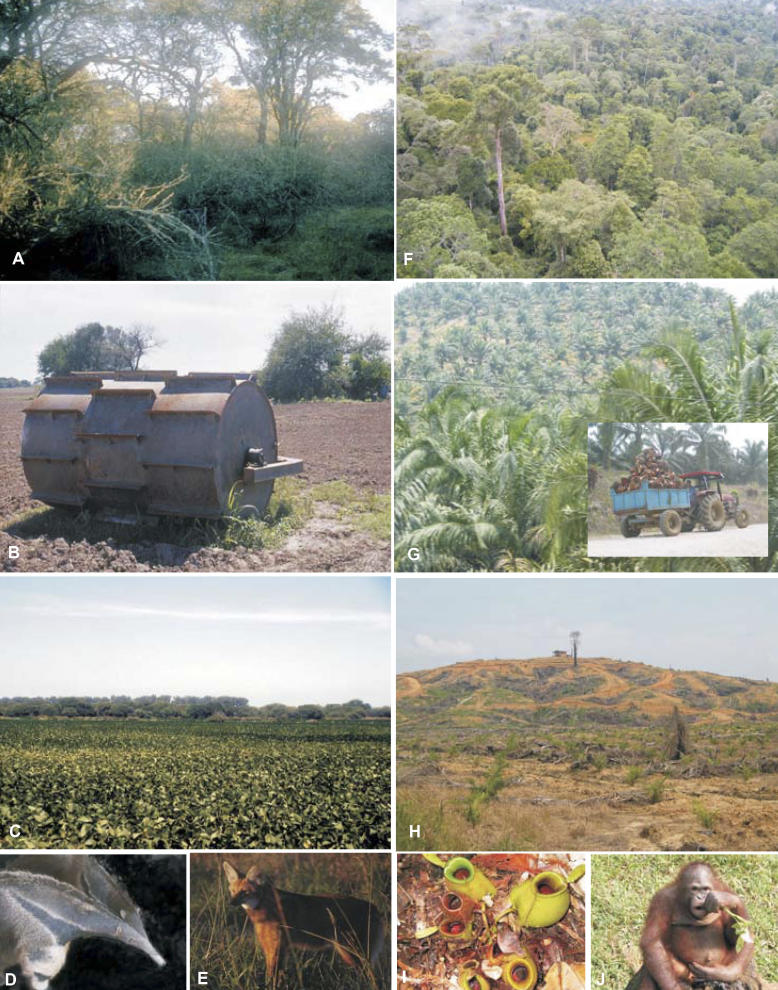
Lost Ecosystem Services and Vanishing Ecological Roles Forest ecosystems in the tropics and subtropics are being quickly replaced by industrial crops and plantations. This provides large amounts of goods for national and international markets, but results in the loss of crucial ecosystem services mediated by ecological processes. In Argentina and Bolivia, the Chaco thorn forest (A) is being felled at a rate considered among the highest in the world (B), to give way to soybean cultivation (C). In Borneo, the Dypterocarp forest, one of the species-richest in the world (F), is being replaced by oil palm plantations (G). These changes are irreversible for all practical purposes (H). Many animal and plant populations have been dramatically reduced by changing land use patterns, to the point that they could be considered functionally extinct, such as the maned wolf (D) and the giant anteater in the Chaco plains (E), and the orangutan (I) and several species of pitcher plants (J) in the Bornean rainforest. Photos by Sandra Díaz, except (A and C), courtesy by Marcelo R. Zak.

Another key message is that, precisely because ecosystem processes depend on the presence and abundance of organisms with particular functional traits, there is wide variation in how ecosystem services—that in turn depend on ecosystem processes—respond to changes in species number as particular species are lost from or get established in the system. So, to the question of how biodiversity matters to ecosystem services, we have to reply that it depends on what organisms there are. Daunting? Certainly, but not hopeless. We know from recent assessments [ 1, 2, 7, 8] that global biodiversity loss is not occurring at random. As a consequence of global change drivers, such as climate, biological invasions, and especially land use, not only is the total number of species on the planet decreasing, but there are also losers and winners. On average, the organisms that are losing out have longer lifespans, bigger bodies, poorer dispersal capacities, more specialized resource use, lower reproductive rates, and other traits that make them more susceptible to human activities such as nutrient loading, harvesting, and biomass removal by burning, livestock grazing, ploughing, clear-felling, etc. A small number of species with the opposite characteristics are becoming increasingly dominant around the world ( Figure 3). Because there are well-established links between functional traits of locally abundant organisms and ecosystem processes, especially for plants [ 9–12], it may become possible to identify changes in ecosystem processes and in ecosystem services that depend on them under different biodiversity scenarios.

## What We Do Not Know: Cascades, Surprises, and Megadiversity Hot-Spots

Some ecosystem services show a saturating relationship to species number—that is, the ecosystem-service response to additional species is large at low number of species and becomes asymptotic beyond a certain number of species. We seldom know what this threshold number is, but we suspect it differs among ecosystems, trophic levels, and services. The experimental evidence indicates that, in the case of primary production (e.g., for plant-based agricultural products), nutrient retention (which can reduce nutrient pollution and sustain production in the long term), and resistance to invasions (which incur damage and control costs in agricultural and other settings) by temperate, herbaceous communities, responses often do not show further significant increases beyond about ten plant species per square meter [ 3, 13]. But in order to achieve this number in a single square meter, a much higher number of species is needed at the landscape level [ 14]. What about slow-growing natural communities, or communities that consist of plant species with more contrasting biology? What about communities that typically include many more species—for example, the megadiverse forest hot-spots of the Amazon and Borneo, where species number can exceed 100 tree species per hectare [ 15]? To what extent are all those species essential for the maintenance of different ecosystem processes and services? Ecological theory [ 16] and traditional knowledge [ 17, 18] suggest that a large number of resident species per functional group, including those species that are rare, may act as ‘insurance” that buffers ecosystem processes and their derived services in the face of changes in the physical and biological environment (e.g., precipitation, temperature, pathogens), but these ideas have yet to be tested experimentally, and no manipulative experiment has been performed in any megadiversity hot-spot.

Most of the links between biodiversity and ecosystem services summarized in Table 1 emerged from theory and manipulative experiments, involved biodiversity within a single trophic level (usually plants), and operated mostly at the level of local communities. However, the most dramatic examples of effects of small changes in biodiversity on ecosystem services have occurred at the landscape level and have involved alterations of food-web diversity through indirect interactions and trophic cascades. Most of these have been “natural experiments,” that is, the unintended consequence of intentional or accidental removal or addition of certain predator, pathogen, herbivore, or plant species to ecosystems. These “ecological surprises” usually involve disproportionately large, unexpected, irreversible, and negative alterations of ecosystem processes, often with repercussions at the level of ecosystem services, with large environmental, economic, and cultural losses. Examples include the cascading effects of decreases in sea otter population that led to coastal erosion in the North Pacific [ 19], and a marked decrease in grassland productivity and nutritional quality in the Aleutian islands as a consequence of decreased nutrient flux from the sea by the introduction of Arctic foxes [ 20] (see [ 3] for a comprehensive list of examples). The vast literature on biological invasions and their ecological and socio-economic impacts [ 21] further illustrates this point. Ecological surprises are difficult to predict, since they usually involve novel interactions among species. They most often result from introductions of predators, herbivores, pathogens and diseases, although cases involving introduced plants are also known. They do not depend linearly on species number or on well-established links between the functional traits of the species in question and putative ecosystem processes or services [ 3, 22].

## Uneven Impacts: Biodiversity and Vulnerable Peoples

People who rely most directly on ecosystem services, such as subsistence farmers, the rural poor, and traditional societies, face the most serious and immediate risks from biodiversity loss. First, they are the ones who rely the most on the “safety net” provided by the biodiversity of natural ecosystems in terms of food security and sustained access to medicinal products, fuel, construction materials, and protection from natural hazards such as storms and floods [ 4]. In many cases the provision of services to the most privileged sectors of society is subsidized but leaves the most vulnerable to pay most of the cost of biodiversity losses. These include, for example, subsistence farmers in the face of industrial agriculture [ 23] and subsistence fishermen in the face of intensive commercial fishing and aquaculture [ 24]. Second, because of their low economic and political power, the less privileged sectors cannot substitute purchased goods and services for the lost ecosystem benefits and they typically have little influence on national policy. When the quality of water deteriorates as a result of fertilizer and pesticide loading by industrial agriculture, the poor are unable to purchase safe water. When protein and vitamins from local sources, such as hunting and fruit, decrease as a result of habitat loss, the rich can still purchase them, whereas the poor cannot. When the capacity of natural ecosystems to buffer the effects of storms and floods is lost because of coastal development [ 25], it is usually the people who cannot flee—for example, subsistence fishermen—who suffer the most. In summary, the loss of biodiversity-dependent ecosystem services is likely to accentuate inequality and marginalization of the most vulnerable sectors of society, by decreasing their access to basic materials for a healthy life and by reducing their freedom of choice and action. Economic development that does not consider effects on these ecosystem services may decrease the quality of life of these vulnerable populations, even if other segments of society benefit. Biodiversity change is therefore inextricably linked to poverty, the largest threat to the future of humanity identified by the United Nations. This is a sobering conclusion for those who argue that biodiversity is simply an intellectual preoccupation of those whose basic needs and aspirations are fulfilled.

## Future Directions

Most of the concrete actions to slow down biodiversity loss fall under the domain of policy making by governments and the civil society. However, the scientific community still needs to fill crucial knowledge gaps. First, we need to know more about the links between biodiversity and ecosystem services in species-rich ecosystems dominated by long-lived plants. Second, if we are to anticipate and avoid undesirable ecological surprises, better models and more empirical evidence are needed on the links between ecosystem services and interactions among different trophic levels. Third, we need to reinforce the systematic screening for functional traits of organisms likely to have ecosystem-level consequences. In this sense, our knowledge of how the presence and local abundance of organisms (especially plants) bearing certain attributes affect ecosystem processes has made considerable progress in the past few years. However, we know much less of how the range of responses to environmental change among species affecting the same ecosystem function contributes to the preservation of ecosystem processes and services in the face of environmental change and uncertainty [ 16, 26]. This is directly relevant to risk assessment of the sustained provision of ecosystem services. Fourth, experimental designs for studying links between biodiversity and ecosystem processes and services need to not only meet statistical criteria but also mimic biotic configurations that appear in real ecosystems as a result of common land-use practices (e.g., primary forest versus monospecific plantations versus enrichment planting, or grazing-timber agroforestry systems versus a diverse grazing megafauna versus a single grazer such as cattle). In pursuing this, traditional knowledge systems and common management practices provide a valuable source of inspiration to develop new designs and testable hypotheses [ 27, 28]. Finally, in order to assist policy decisions and negotiation among different local, national, and international stakeholders, considerable advance is needed in the evaluation and accounting of ecosystem services [ 29, 30]. The challenge here is to find ways to identify and monitor services that are as concrete as possible, but at the same time not alienate the view of less powerful social actors or bias the analysis against services that are difficult to quantify or grasp.

## The Bottom Line

By affecting the magnitude, pace, and temporal continuity by which energy and materials are circulated through ecosystems, biodiversity in the broad sense influences the provision of ecosystem services. The most dramatic changes in ecosystem services are likely to come from altered functional compositions of communities and from the loss, within the same trophic level, of locally abundant species rather than from the loss of already rare species. Based on the available evidence, we cannot define a level of biodiversity loss that is safe, and we still do not have satisfactory models to account for ecological surprises. Direct effects of drivers of biodiversity loss (eutrophication, burning, soil erosion and flooding, etc.) on ecosystem processes and services are often more dramatic than those mediated by biodiversity change. Nevertheless, there is compelling evidence that the tapestry of life, rather than responding passively to global environmental change, actively mediates changes in the Earth's life-support systems. Its degradation is threatening the fulfillment of basic needs and aspiration of humanity as a whole, but especially, and most immediately, those of the most disadvantaged segments of society.

**Box 1. From Ecosystem Processes to Human Well-Being**

**Ecosystem processes** are intrinsic processes and fluxes whereby an ecosystem maintains its integrity (such as primary productivity, trophic transfer from plants to animals, decomposition and nutrient cycling, evapotranspiration, etc.). They exist independently from human valuation, and their magnitude and rate can be established regardless of the cultural, economic, and social values and interests of different human groups ( Figure 1, Ecosystem Processes box).

**Ecosystem services** are the benefits provided by ecosystems that contribute to making human life both possible and worth living. Ecosystem services are context-dependent; that is, the same ecosystem process can produce an ecosystem service that is highly valued by one society or stakeholder group but not highly valued by other societies or groups. Some ecosystem services involve the direct provision of material and non-material goods and are associated directly with the presence of particular species of plants and animals—for example, food, timber, medicines, and ritual materials ( Figure 1, path 1 and bottom sub-box of Ecosystem Services box). Other ecosystem services arise, either directly or indirectly, from the continued functioning of ecosystem processes. For example, the service of formation, retention, and sustained fertility of soils necessary for the production of plants and animals considered important by different human societies depends on the ecosystem processes of decomposition, nutrient cycling by soil microbiota, and the retention of water and soil particles by a well-developed root network ( Figure 1, path 2 and top sub-box in red of Ecosystem Services box). Some authors (e.g., [ 30]) have advocated a stricter definition of ecosystem services as components of nature that are directly enjoyed, consumed, or used in order to maintain or enhance human well-being. Although such an approach can be useful when it comes to ecosystem service accounting, our emphasis here is conceptual, and therefore we prefer to use the broader, widely accepted definitions and classification adopted by the Millennium Ecosystem Assessment [ 4]. This is because some ecosystem services (e.g., food provision) can be quantified in units that are easily comprehensible by policy makers and the general public. Others—for example, the services that regulate and support the production of tradable goods—are more difficult to quantify. If a criterion based on economic accounting is applied too strictly, there is a risk that ecosystem service assessment could be biased toward services that are easily quantifiable, but not necessarily the most critical ones [ 29].

**Human well-being** is a human experience that includes the basic materials for a good life, freedom of choice and action, health, good social relationships, a sense of cultural identity, and a sense of security. The sense of well-being is strongly dependent on the specific cultural, geographical, and historical context in which different human societies develop, and is determined by cultural-socioeconomic processes as well as by the provision of ecosystem services. However, the well-being of the vast majority of human societies is based more or less directly on the sustained delivery of fundamental ecosystem services, such as the production of food, fuel, and shelter, the regulation of the quality and quantity of water supply, the control of natural hazards, etc. (see Figure 1, path 3).
